# Supplementary material for: Local Scale Exposure and Fate of Engineered Nanomaterials
Source: Toxics. 2022 Jun 29;10(7):354. doi: 10.3390/toxics10070354 (PMC9319542; doi:10.3390/toxics10070354)
Supplement: Supplementary file 1 [file toxics-10-00354-s001.zip › toxics-1737819-supplementary.pdf]

Supplementary Materials: Local Scale Exposure and Fate of Engineered Nanomaterials

Mikko Poikkimäki, Joris T.K. Quik, Arto Säämänen and Miikka Dal Maso

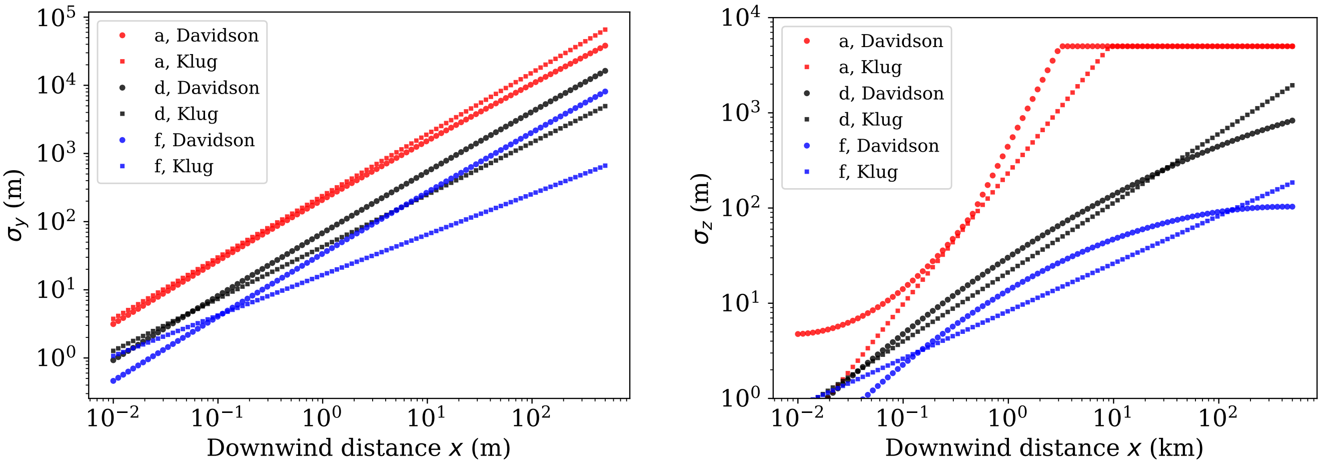

Figure S1. Crosswind σ_y and vertical σ_z dispersion parameters for different atmospheric stability classes calculated by parametrizations of Davidson and Klug.

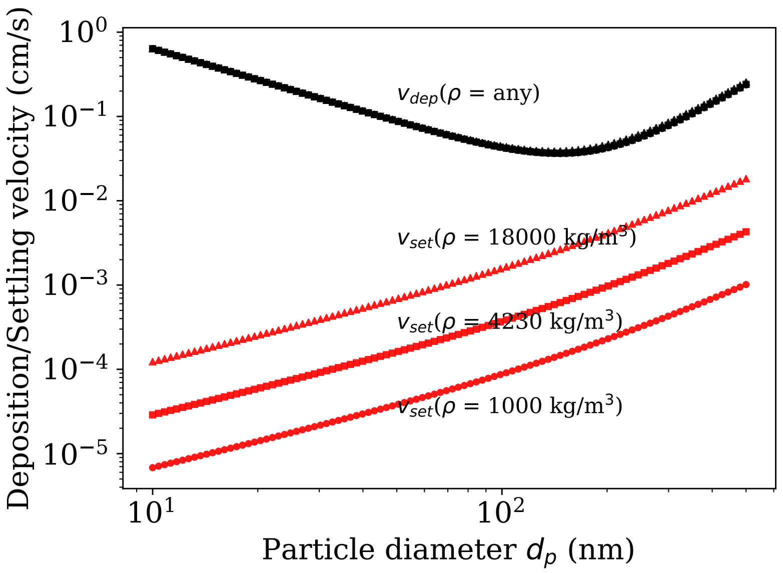

Figure S2. Deposition (v_{dep}) and settling velocities (v_{set}) as a function of particle diameter for different particle densities ρ calculated with the algorithm of Rannik et al. [1].

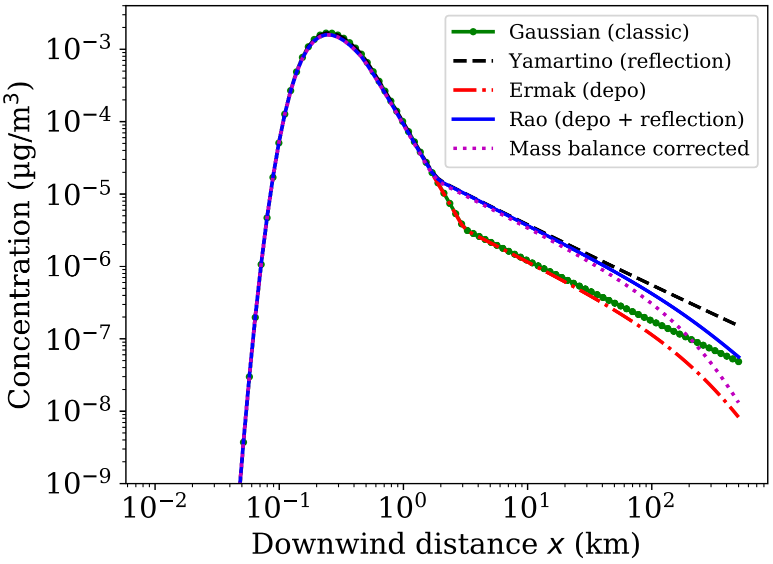

Figure S3. A comparison of ground level concentrations for five different Gaussian formulas: classic Gaussian, Yamartino with boundary layer reflection, Ermak with deposition, Rao with deposition and reflection as well as mass balance corrected concentration used in this study. Input parameters: Atmospheric stability class a, boundary layer height 2 km, wind speed 10 m/s, source height 50 m, deposition velocity 1 cm/s and Davidson's dispersion parametrization.

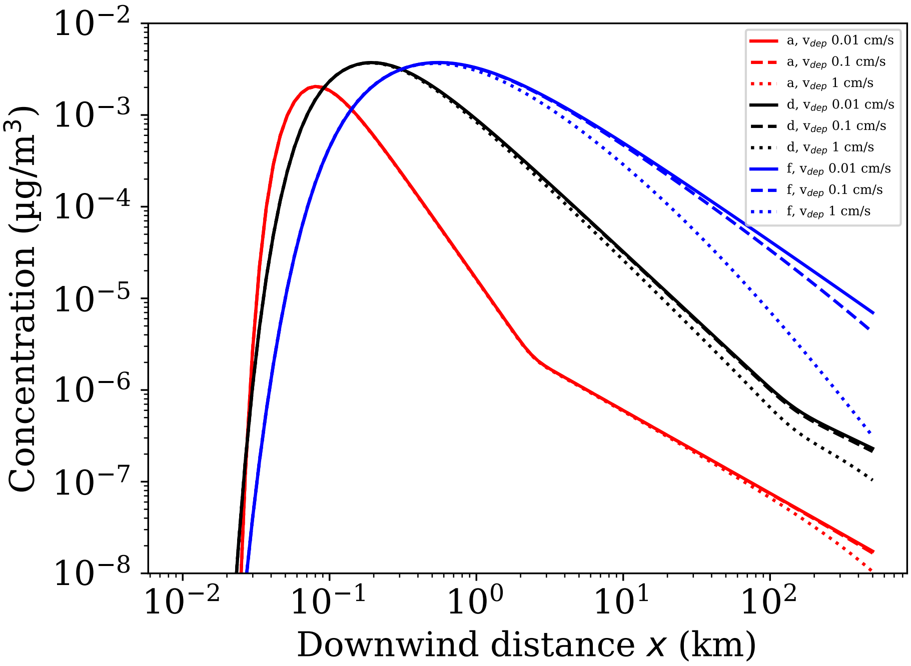

Figure S4. Ground level concentration (mass balance corrected) at the centerline of the plume as a function of downwind distance for different deposition velocities and different atmospheric stability classes (a, d, f). Input parameters: Boundary layer height 1 km, wind speed 10 m/s, source height 10 m and Klug's dispersion parametrization.

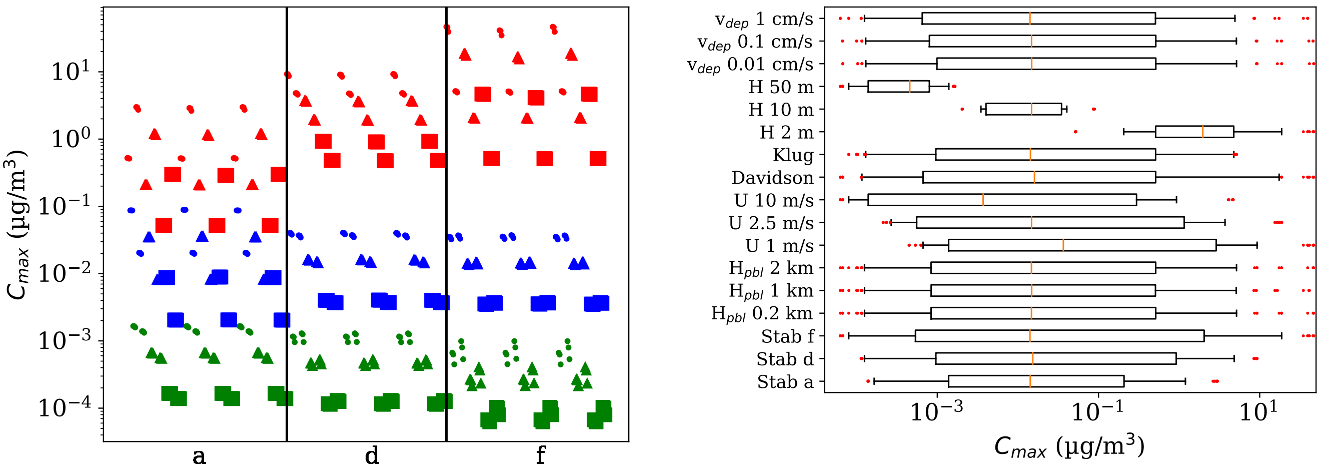

Figure S5. Maximum ground level concentrations for a generic pollutant. (**a**) All data points grouped for the atmospheric stability classes (a, d, f). Small circles correspond to the the wind speed of 1 m/s, triangles the speed of 2.5 m/s and large squares the speed of 10 m/s. Red markers have a source height of 2 m, blue a height of 10 m and green a height of 50 m. (**b**) Sensitivity of the input parameters. The yellow line represents the median value of all observations for a certain parameter value. The boxes present 25 and 75% quartiles, while the whiskers correspond to the 5th and 95th percentiles. Red dots are outliers.

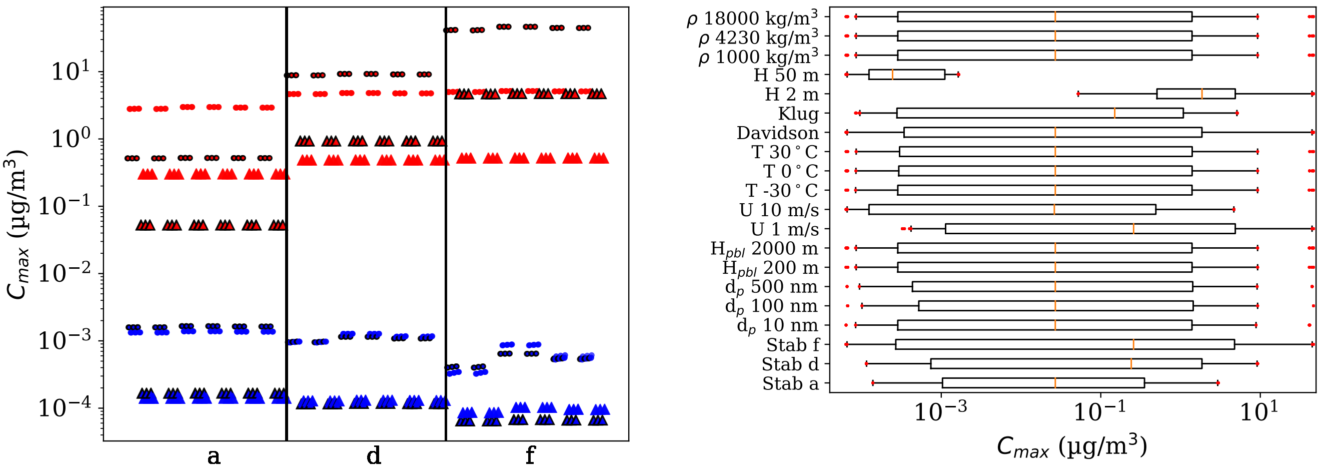

Figure S6. Maximum ground level concentrations for ENM simulations. (**a**) All data points grouped for the atmospheric stability classes (a, d, f). Small circles correspond to the wind speed of 1 m/s and large triangles the speed of 10 m/s. Red markers correspond to the source height of 2 m and blue the height of 50 m. The black marker edges coincide with Davidson's dispersion parametrization, whereas Klug's parametrization has no edge color. (**b**) Sensitivity of the input parameters. The yellow line represents the median value of all observations for a certain parameter value. The boxes present 25 and 75% quartiles, while the whiskers correspond to the 5th and 95th percentiles. Red dots are outliers.

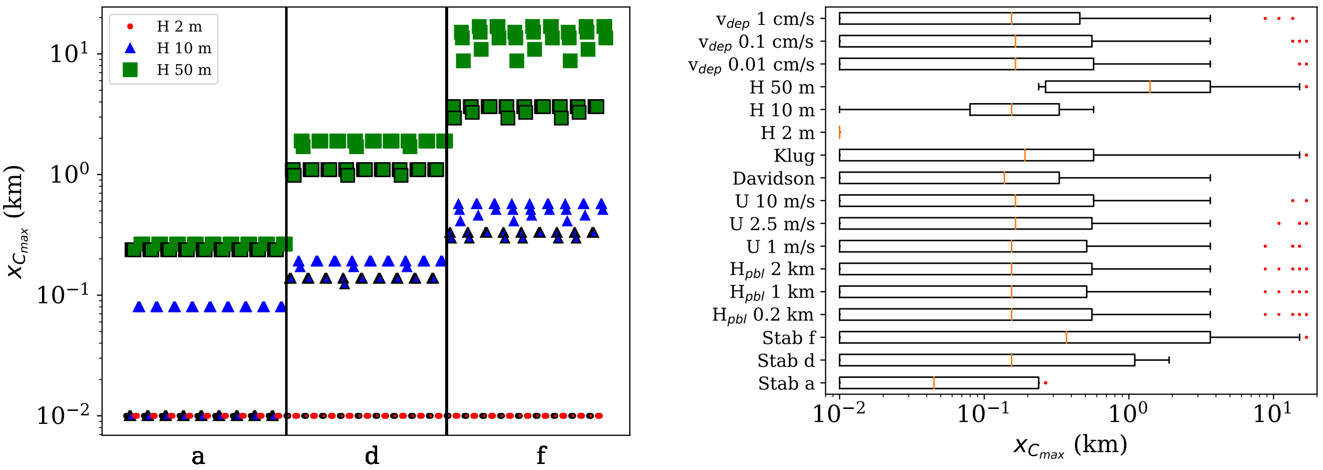

Figure S7. Distances at which maximum ground level concentrations are reached for a generic pollutant. (**a**) All data points grouped for the atmospheric stability classes (a, d, f) for three different source heights. The black marker edges coincide with Davidson's dispersion parametrization, whereas without edge color have Klug's parametrization. (**b**) Sensitivity of the input parameters. The yellow line represents the median value of all observations for a certain parameter value. The boxes present 25 and 75% quartiles, while the whiskers correspond to the 5th and 95th percentiles. Red dots are outliers.

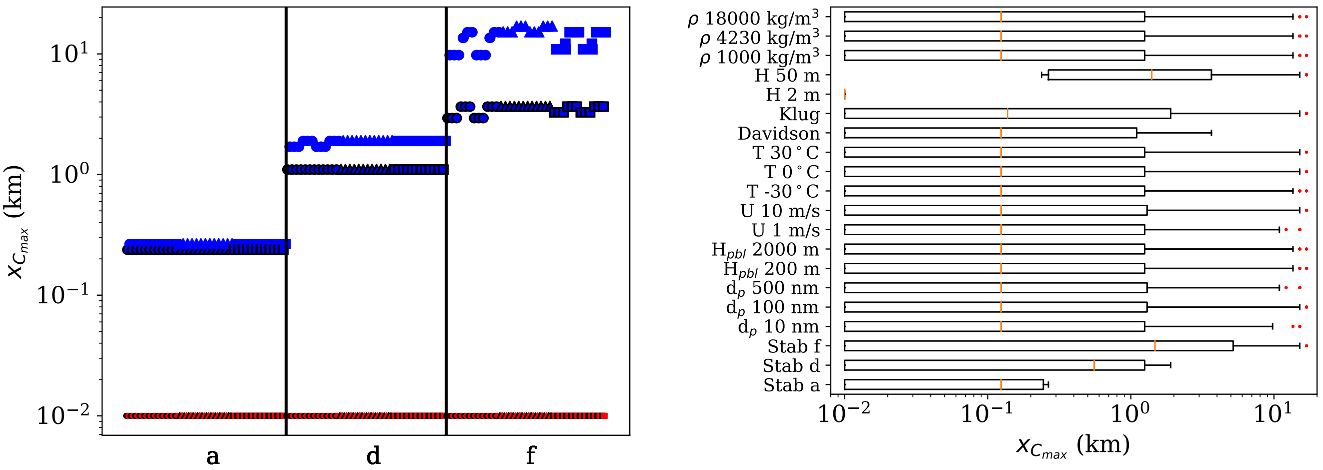

Figure S8. Distances at which maximum ground level concentrations are reached for ENMs. (**a**) All data points grouped for the atmospheric stability classes (a, d, f). Circles correspond to the particle size of 10 nm, triangles the size of 100 nm and squares the size of 500 nm. Red markers have a source height of 2 m and blue a height of 50 m. The black marker edges coincide with Davidson's dispersion parametrization, whereas without edge color have Klug's parametrization. (**b**) Sensitivity of the input parameters. The yellow line represents the median value of all observations for a certain parameter value. The boxes present 25 and 75% quartiles, while the whiskers correspond to the 5th and 95th percentiles. Red dots are outliers.

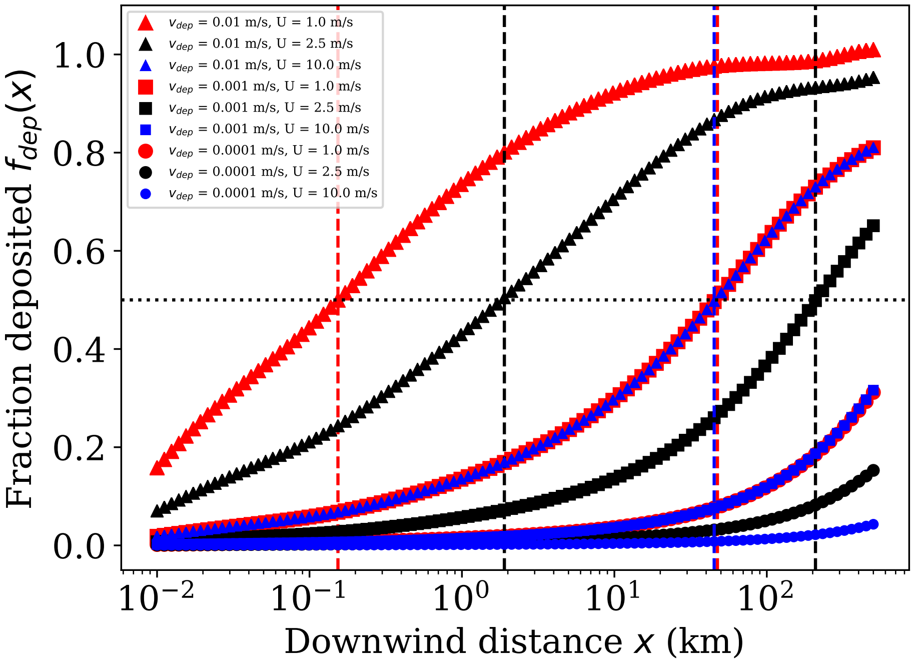

Figure S9. Fraction of aerosol deposited to the ground for different deposition velocities v_{dep} and wind speeds U. Vertical dashed lines represent the distances of 50 percent deposited, i.e., x_{50} distances.

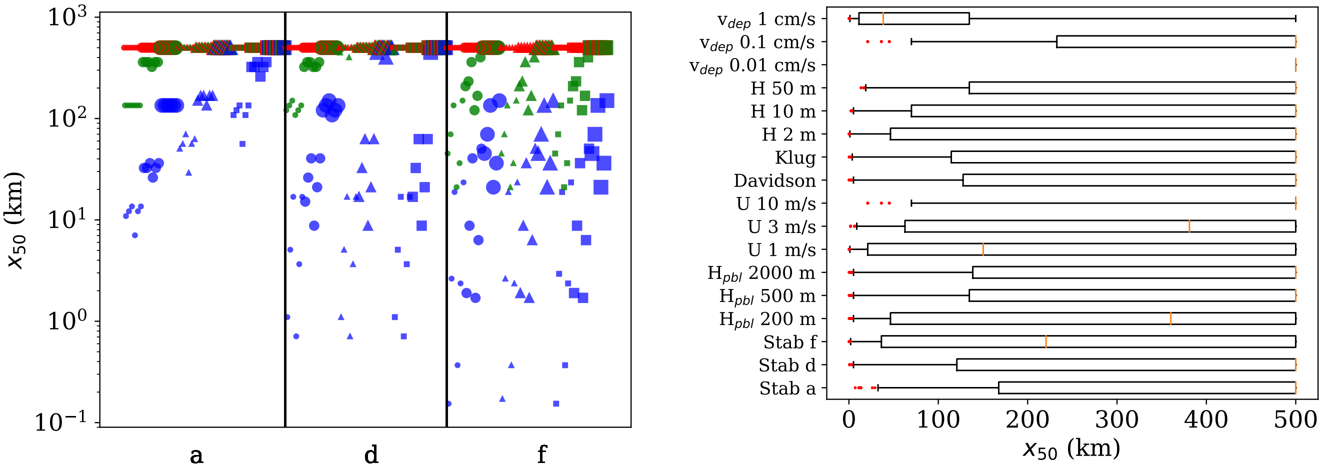

Figure S10. x_{50} distances for the simulations of a generic pollutant. (**a**) All data points grouped for the atmospheric stability classes (a, d, f). Circles correspond to the boundary layer height of 0.2 km, triangles the height of 1 km and squares the height of 2 km. Red markers have a deposition velocity of 0.01 cm/s, green a velocity of 0.1 cm/s and blue a velocity of 1 cm/s. Smallest marker sizes represent the wind speed of 1 m/s, medium sizes the speed of 2.5 m/s and largest markers the speed of 10 m/s. (**b**) Sensitivity of the input parameters. The yellow line represents the median value of all observations for a certain parameter value. The boxes present 25 and 75% quartiles, while the whiskers correspond to the 5th and 95th percentiles. Red dots are outliers.

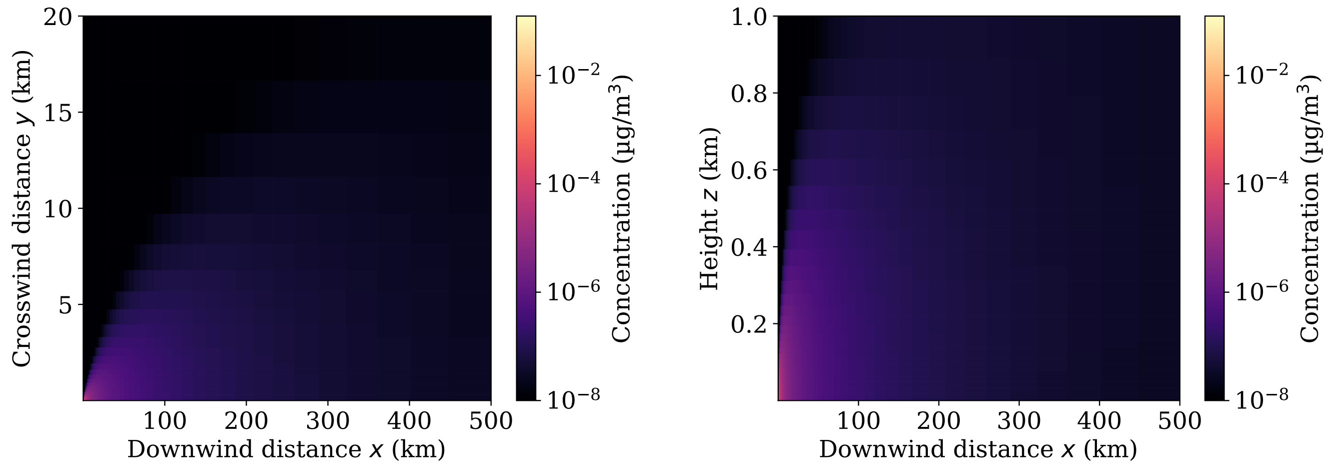

Figure S11. Airborne ENM concentration as a function of (**a**) downwind and crosswind distances at ground level (2 m) as well as (**b**) downwind distance and height from ground in the centerline of the plume. Simulation of the case study of Koivisto et al. [2] for atmospheric stability class d.

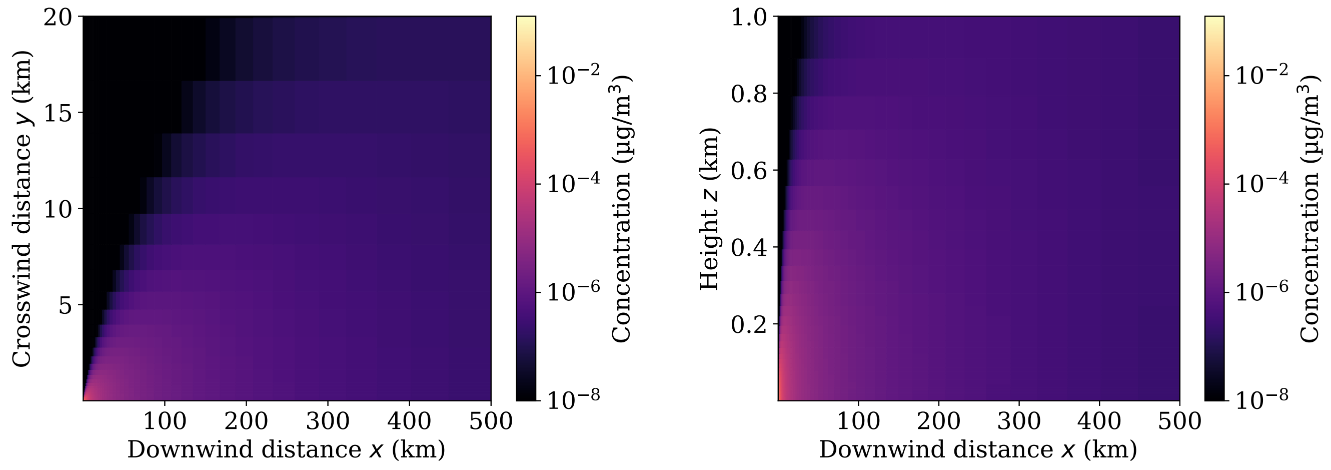

Figure S12. Airborne ENM concentration as a function of (**a**) downwind and crosswind distances at ground level (2 m) as well as (**b**) downwind distance and height from ground in the centerline of the plume. Simulation of the case study of Fonseca et al. [3] for atmospheric stability class d.

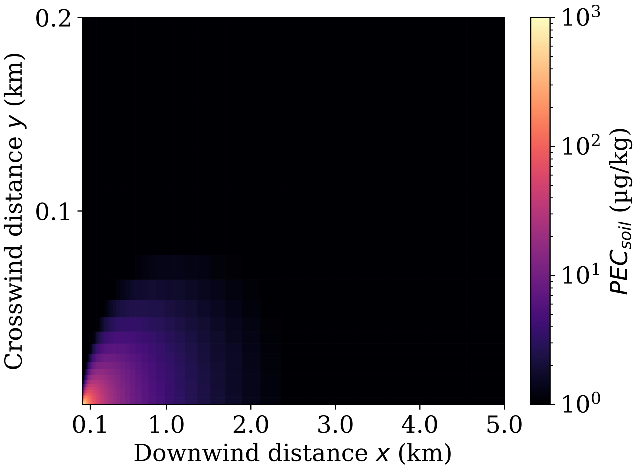

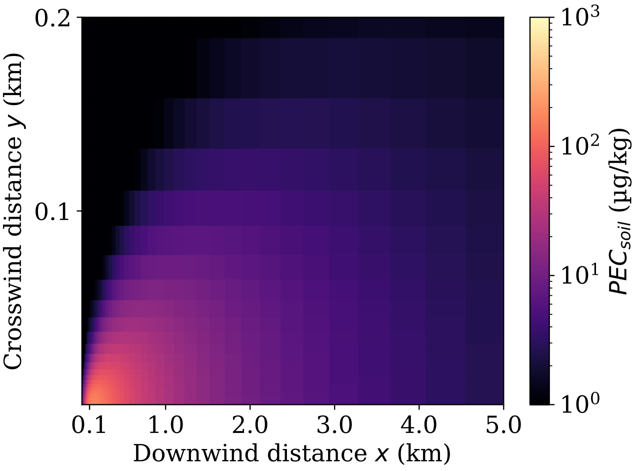

Figure S13. Predicted environmental concentration of nano-TiO_2 in soil (PEC_{soil}) at different cross- and downwind distances from the source after 30 years of continuous emission from (**a**) a spray coating facility Koivisto et al. [2] and (**b**) a paint factory Fonseca et al. [3]. Black area represents concentrations below 1 µg/kg.

Table S1. SimpleBox4nano model parameter values employed in the case study comparison to the dispersion model developed in this study (ADiDeNano). We report here only the values changed from SB4N default values on the input sheet.

**Parameter**	**Value**
Substance	nTiO2_P25_CaLIBRAte_D6_3
Scenario	nTiO2_P25_CaLIBRATE_D6_4
Case	CaLIBRAte nTiO2 p25 case
Radius primary ENP	260 nm
Density primary ENP	940 kg/m^3
Reg. Emission to air	0.0009 t/yr
Reg. Emission to lake water	0 t/yr
Reg. Emission to fresh water	0 t/yr
Reg. Emission to agricultural soil	0 t/yr
Reg. Emission to other soil	0 t/yr
Cont. Emission to air	0 t/yr
Cont. Emission to lake water	0 t/yr
Cont. Emission to fresh water	0 t/yr
Cont. Emission to agricultural soil	0 t/yr
Cont. Emission to other soil	0 t/yr
**Regional and Continental**	
Temperature	15 °C
Wind speed	2.5 m/s
Average precipitation	0.00001 mm/yr

References

1. Rannik, Ü.; Aalto, P.; Keronen, P.; Vesala, T.; Kulmala, M. Interpretation of aerosol particle fluxes over a pine forest: Dry deposition and random errors. *Journal of Geophysical Research* **2003**, *108*, 4544. doi:10.1029/2003JD003542.
2. Koivisto, A.J.; Del Secco, B.; Trabucco, S.; Nicosia, A.; Ravegnani, F.; Altin, M.; Cabellos, J.; Furxhi, I.; Blosi, M.; Costa, A.; et al. Quantifying Emission Factors and Setting Conditions of Use According to ECHA Chapter R. 14 for a Spray Process Designed for Nanocoatings—A Case Study. *Nanomaterials* **2022**, *12*, 596. doi:10.3390/nano12040596.
3. Fonseca, A.S.; Viitanen, A.K.; Kanerva, T.; Säämänen, A.; Aguerre-Chariol, O.; Fable, S.; Dermigny, A.; Karoski, N.; Fraboulet, I.; Koponen, I.K.; et al. Occupational exposure and environmental release: the case study of pouring TiO2 and filler materials for paint production. *International Journal of Environmental Research and Public Health* **2021**, *18*, 418. doi:10.3390/ijerph18020418.
